# Unusual Transalveolar and Transmuco-Gingival Root Avulsion of a Fractured Primary Central Incisor: A Case with an 8-Year Follow-Up

**DOI:** 10.1155/2015/914846

**Published:** 2015-02-22

**Authors:** E. Ferrés-Amat, C. Díaz-Martínez, S. Herrera-Martínez, I. Maura-Solivellas, E. Ferrés-Padró

**Affiliations:** ^1^Pediatric Dentistry Service and Oral and Maxillofacial Surgery Service, Fundació Hospital de Nens de Barcelona, Consell de Cent 437, 08009 Barcelona, Spain; ^2^Department of Oral and Maxillofacial Surgery, Faculty of Dentistry, Universitat Internacional de Catalunya, Barcelona, Spain; ^3^Service of Pediatric Dentistry, Fundació Hospital de Nens de Barcelona, Barcelona, Spain; ^4^Service of Oral and Maxillofacial Surgery, Fundació Hospital de Nens de Barcelona, Barcelona, Spain

## Abstract

The purpose of this unique case report is to describe a very unusual dentoalveolar fracture associated with avulsion of the near-complete root. A 3-year-old male patient came for consultation after a dentoalveolar trauma with a “fragment that looks like canine” found in his mouth by his mother. This boy suffered root fracture of the upper primary central right incisor, accompanied by transalveolar and transmuco-gingival avulsion of the tooth root fragment, leaving the crown in its position in the dental arch. Clinical and radiological examinations were performed in order to follow up the case: 15 days, one month, and three months after trauma, the crown had a slight mobility without other clinical or radiological signs. After six months, the upper primary central right incisor's crown was exfoliated. Open bite due to the persistence of the pacifier habit favored the crown retention in the mouth. This case emphasizes the importance of primary diagnosis and follow-up of trauma cases. To the best of our knowledge, this kind of dental injury has not been previously described in the literature nor in the current Dental Trauma guidelines for the management of traumatic dental injuries in the primary dentition.

## 1. Introduction

Dental traumas are the second cause of pediatric dental care, after dental caries, although its incidence is increasing. In the primary dentition, many accidents usually occur during the first three years of life, because it is during this period when the child moves from a state of total dependence in his or her movements to a relative stable situation, as he or she learns to bend down, crawl, stand, and walk [1–4]. The most common reason of dental trauma in preschool children was reported to be falls [3–15]. Most accidents involving the primary dentition take place in a domestic environment [6, 14].

There is a similar prevalence in both sexes [3–6]. Many studies report that the gender differences are statistically nonsignificant in primary dentition [11–14].

The maxillary arch is more affected than the mandibular arch [16]. The upper front teeth are the most commonly involved because of their exposed position in the dental arch [6–16]. The maxillary incisors and, more specifically the central incisors, are the most commonly injured teeth [1–7].

The literature reports luxations and avulsions as the most frequent types of trauma on the deciduous dentition rather than hard tissue injuries in primary dentition. This is due to higher elasticity of the bone and relatively short roots of small children that may favour luxation injuries [12–18].

A root fracture is a fracture confined to the root of the tooth involving cementum, dentin, and the pulp. The periodontium is also damaged and the coronal fragment is displaced or avulsed. The diagnosis is usually clinical and radiological. Sometimes radiological diagnosis of the fracture can be difficult [16–19].

An avulsion is the complete displacement of the tooth out of its socket. It often affects central incisors in both dentitions, and, in most cases, only a single tooth is involved [19–21].

This case report is about a transmuco-gingival and transalveolar fractured root avulsion, which means that it is a partial avulsion.

The aim of this first case report is to present a unique dental injury, which has not been previously described in the literature or in the current Dental Trauma guidelines for the management of traumatic dental injuries in the primary dentition.

## 2. Case Presentation

This is a case of a 3-year-old male patient, with no medical or dental history of interest, suffered a dental trauma after a domestic accident and went as an emergency to the Pediatric Dentistry Service of the Hospital.

Chief complaint: his mother went to the emergency room with the fragment of her child's tooth root in her hand, explaining that she had found this “loose canine in the child's mouth” after a dental trauma. In the physical examination, the following was found: a bruise, abrasion, and laceration of the maxillary buccal mucosa and gingiva of the primary maxillary central incisors zone. There was mobility in the primary maxillary right central incisor and light mobility without displacement in the primary maxillary left central incisor. The clinical and radiological exploration of the lateral incisors was normal, without any pathology. The patient presented with anterior open bite because of pacifier habit persistence.

**Figure 1:**
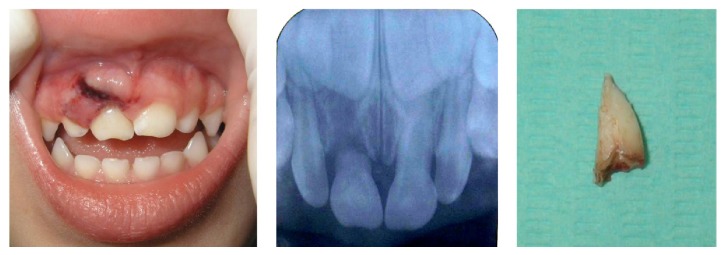
Intraoral view two hours after trauma. Periapical radiography. Root of the primary maxillary right central incisor root.

Periapical radiographic examination revealed the near complete absence of the primary maxillary right central incisor root. This exploration provided evidence that the patient had suffered a root fracture of the primary maxillary right central incisor (fracture located to cervical part of the root) accompanied by transalveolar and transmuco-gingival avulsion of the tooth root fragment (Figure 1). The dental crypts of the maxillary alveolar process of the developing permanent teeth were found without any radiological alteration (the dental follicle of the permanent maxillary right central incisor was in 5 Nolla stage).

Root avulsion of the primary maxillary right central incisor, subluxation of the primary maxillary left central incisor, and fracture of the alveolar process were diagnosed.

An antibiotic regimen of amoxicillin-clavulanic acid was started as a treatment for one week. Soft diet was recommended and oral hygiene instructions were explained (brush with a soft brush after every meal). It was also recommended to apply chlorhexidine 0.1% topically to the affected area with cotton swabs twice a day for one week and to restrict the use of a pacifier, as well as to avoid putting pressure on the traumatized area.

Several posttrauma follow-up appointments were performed, exactly 15 days, one month, and three months after the trauma.

During the first examination, 15 days after the trauma, the clinical intraoral examination showed that the buccal mucosa of the primary maxillary right central incisor was healing, and the periapical radiography showed absence of the primary maxillary right central incisor root, but without any alteration or pathological image. The crown of the primary right central incisor did not present mobility (it was in its initial position in the dental arch) and there was not any risk of aspiration. In addition to this, the child had already given up the pacifier habit, as it was recommended in the posttrauma instructions.

Another examination was performed one month after the trauma. The clinical intraoral examination showed normal buccal mucosa and the primary maxillary right central incisor crown did not show mobility. A periapical radiography was also performed.

**Figure 2:**
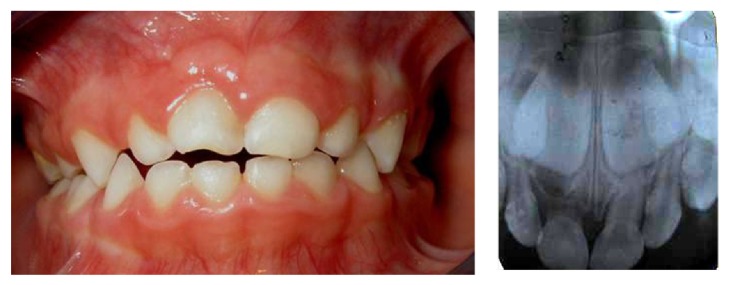
Examination 3 months after the trauma.

Another examination was performed 3 months after the trauma (Figure 2). The clinical intraoral examination showed a perfectly healed vestibular mucosa with normal appearance and mobility of the primary maxillary right central incisor crown. A periapical radiography was also performed.

**Figure 3:**
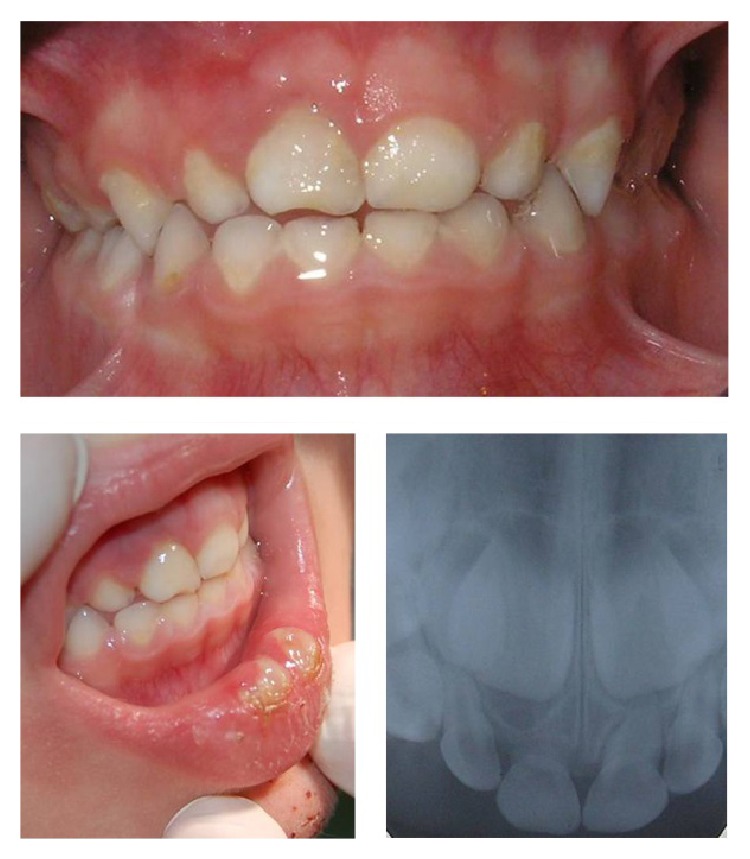
Last trauma.

After the last examination, the patient went to the emergency room three more times because he suffered different traumas on the primary maxillary right central incisor (playing football, hitting himself with a chair, and running). A lacerated contused wound in the lower lip was observed during the last physical trauma examination, as well as an abrasion injury on the chin (Figure 3).

Four months after the trauma, the primary maxillary right central crown remained in its position in the dental arch.

As there was a lack of occlusal contact with the opposing tooth, which would have caused the crown extrusion, the tooth remained in his mouth. This absence of occlusal contact was due to the anterior open bite caused by the former pacifier habit.

**Figure 4:**
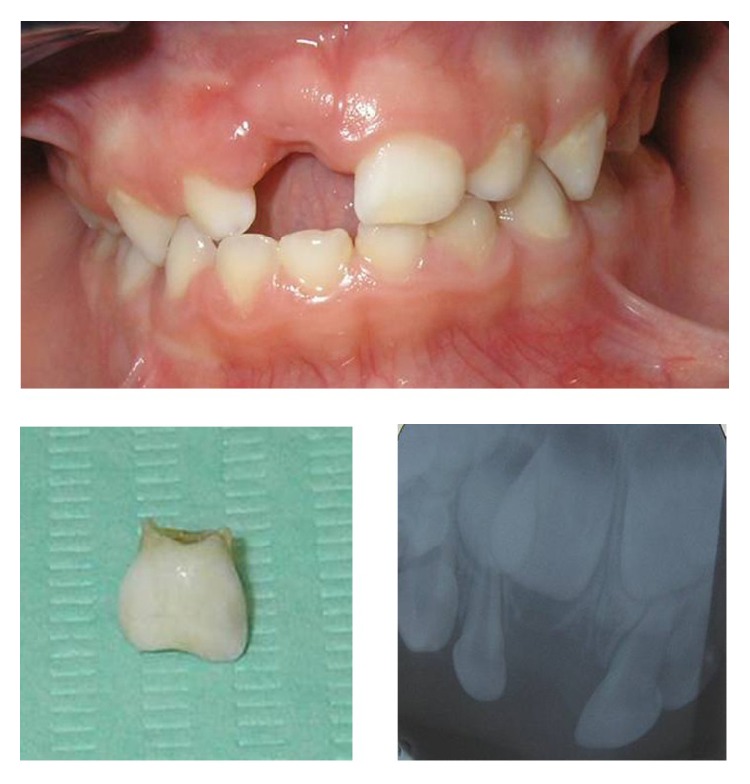
Six months after the trauma.

Six months after the trauma, the patient's mother called to tell us that the primary maxillary right central incisor crown had been exfoliated and we appointed another examination to assess the state of the crown and alveolar bone (Figure 4).

**Figure 5:**
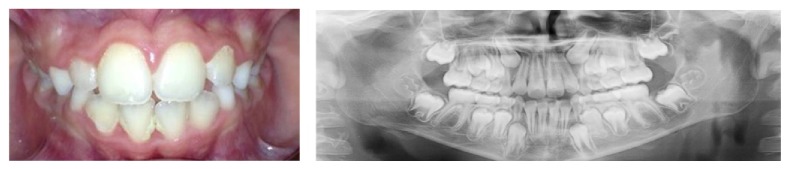
Permanent maxillary incisors normally erupted after an 8-year follow-up.

After the exfoliation of the primary maxillary right central incisor crown, the growth and development of the patient had to be monitored in order to check the eruption of the permanent maxillary incisors. After an 8-year follow-up, the permanent maxillary central incisor had normally erupted (Figure 5).

## 3. Discussion

Dental trauma in primary teeth may cause pain and loss of function and may also affect the development of permanent teeth and occlusion, which may result in physical, emotional, and behavioural problems for the children and their parents or guardians [22, 23].

Most studies show that many children with trauma in primary teeth do not come to the dentist in the first 24 hours after injury [11, 12, 16]. This is taken as an indicator; patients and their parents do not give importance to traumatic dental injuries, having a tendency to attend after a period of time has elapsed or waiting until they had acute symptoms of inflammation and/or aesthetic problems [16].

The time interval between injury and treatment significantly influences the choice of treatment and the prognosis of traumatized teeth [15–22].

The most recommended treatment of traumatic dental injury to primary dentition is examination and monitoring [4, 14, 15, 18]. Posttrauma follow-up appointments are essential for the proper monitoring of the progress of each patient [20, 24, 25].

Radiological examination is essential for diagnosis and control of dentoalveolar trauma damages. To asses a root fracture diagnosis, a clinical and radiological exploration is necessary [10, 20, 22, 24].

According to some reports, the malocclusion is a risk factor for pediatric traumatic dental injury, especially overjet in the anterior incisor region, open bite, and cross-bite [23, 26, 27].

In this particular case the open bite of the patient has promoted the retention of the crown in the mouth, due to the absence of occlusal contact with the opposing tooth. The authors decided to leave a rootless tooth in the mouth due to its unusual clinical situation, which was similar to the rest of temporary tooth, since exfoliation is a spontaneous process in nature.

The clinical reasons why the authors did not perform the crown extraction are the following ones: the absence of the crown mobility and the lack of aspiration risk.

Additionally, the root avulsion through the buccal periodontium (alveolar bone process, mucosa, and gingiva) without injury of the dental follicle inside the dental crypt allowed the normal odontogenesis and the permanent central incisor eruption.

No similar cases to the one that has been described have been found in the literature research. Moreover, this type of unusual fracture is not included in the Dental Traumatology Guidelines classifications.
